# Validation of Six Genetic Determinants of Susceptibility to Estrogen-Induced Mammary Cancer in the Rat and Assessment of Their Relevance to Breast Cancer Risk in Humans

**DOI:** 10.1534/g3.114.011163

**Published:** 2014-05-28

**Authors:** John A. Colletti, Kristin M. Leland-Wavrin, Scott G. Kurz, Maureen Peters Hickman, Nicole L. Seiler, Nyssa Becker Samanas, Quincy A. Eckert, Kirsten L. Dennison, Lina Ding, Beverly S. Schaffer, James D. Shull

**Affiliations:** * McArdle Laboratory for Cancer Research, Department of Oncology, UW Carbone Cancer Center, School of Medicine and Public Health, University of Wisconsin-Madison, Madison, Wisconsin 53706-1599; † Department of Genetics, Cell Biology, and Anatomy, University of Nebraska Medical Center, Omaha, Nebraska 68198-5805

**Keywords:** ACI rat, Brown Norway rat, quantitative trait locus, estradiol, breast cancer

## Abstract

When treated with 17β-estradiol, female ACI rats (*Rattus norvegicus*) rapidly develop mammary cancers that share multiple phenotypes with luminal breast cancers. Seven distinct quantitative trait loci that harbor genetic determinants of susceptibility to 17β-estradiol−induced mammary cancer have been mapped in reciprocal intercrosses between susceptible ACI rats and resistant Brown Norway (BN) rats. A panel of unique congenic rat strains has now been generated and characterized to confirm the existence of these quantitative trait loci, designated *Emca3* through *Emca9*, and to quantify their individual effects on susceptibility to 17β-estradiol−induced mammary cancer. Each congenic strain carries BN alleles spanning an individual *Emca* locus, introgressed onto the ACI genetic background. Data presented herein indicate that BN alleles at *Emca3*, *Emca4*, *Emca5*, *Emca6*, and *Emca9* reduce susceptibility to 17β-estradiol−induced mammary cancer, whereas BN alleles at *Emca7* increase susceptibility, thereby confirming the previous interval mapping data. All of these *Emca* loci are orthologous to regions of the human genome that have been demonstrated in genome-wide association studies to harbor genetic variants that influence breast cancer risk. Moreover, four of the *Emca* loci are orthologous to loci in humans that have been associated with mammographic breast density, a biomarker of breast cancer risk. This study further establishes the relevance of the ACI and derived congenic rat models of 17β-estradiol−induced mammary cancer for defining the genetic bases of breast cancer susceptibility and elucidating the mechanisms through which 17β-estradiol contributes to breast cancer development.

Breast cancer remains the second-leading cause of cancer-related mortality for women in the United States despite significant improvements in prevention, diagnosis, and treatment over the past two decades. The etiology of breast cancer is complex and incompletely understood. A family history of breast cancer and/or ovarian cancer is among the strongest known risk factors. Approximately 5–10% of breast cancers result from inheritance of rare, but highly to moderately penetrant, mutations in a small number of well-studied tumor suppressor genes, including *BRCA1* and *BRCA2* (Ponder *et al.* 2005). More recently, genome-wide association studies (GWAS) have localized within the human genome more than 70 common genetic variants that act as low-penetrance determinants of breast cancer risk, and together these variants are estimated to explain approximately 15% of heritable risk (Easton *et al.* 2007; Stacey *et al.* 2008; Thomas *et al.* 2009; Turnbull *et al.* 2010; Siddiq *et al.* 2012; Garcia-Closas *et al.* 2013; Ghoussaini *et al.* 2013; Michailidou *et al.* 2013). The identities as well as the sites and mechanisms of action of the causal genetic variants mapped in GWAS have not been defined.

Numerous laboratory, clinical, and population-based studies implicate endogenous and exogenous estrogens in breast cancer etiology. For example, use of selective estrogen receptor modulators, such as tamoxifen and raloxifene, has been shown to reduce dramatically the incidence of breast cancer in women who are at a high risk for developing the disease (Umar *et al.* 2012; Den Hollander *et al.* 2013). Similarly, aromatase inhibitors, which block estrogen production by inhibiting the aromatization of androgen precursors, also have been demonstrated to reduce the incidence of breast cancer in high-risk populations (Umar *et al.* 2012; Den Hollander *et al.* 2013). Conversely, the use of hormone-replacement regimens by postmenopausal women has been strongly associated with an increased risk of breast cancer (Narod 2011).

We are using the ACI rat model of 17β-estradiol (E2)-induced mammary cancer in genetic studies to map and identify genetic variants that determine breast cancer risk as well as to define more fully the mechanisms through which estrogens contribute to breast cancer development. Female ACI rats are uniquely susceptible to mammary cancer when treated with physiological levels of E2 (Shull *et al.* 1997; Spady *et al.* 1998; Shull *et al.* 2001). The mammary cancers that develop in E2-treated ACI rats express estrogen receptor-alpha and progesterone receptor, are dependent upon estrogens for survival and growth, and exhibit genome instability (Harvell *et al.* 2000; Adamovic *et al.* 2007; Ruhlen *et al.* 2009). Each of these tumor phenotypes is also a feature of luminal type breast cancers in humans. Interval mapping studies that use F2 progeny generated in intercrosses between susceptible ACI rats and resistant Copenhagen (COP) or Brown Norway (BN) rats revealed the locations of nine quantitative trait loci (QTL), designated *Emca1* (*Estrogen-induced mammary cancer*) through *Emca9*, that influence susceptibility to E2-induced mammary cancer (Gould *et al.* 2004; Schaffer *et al.* 2006; Shull 2007; B. Schaffer and J. Shull, unpublished data). As data from GWAS continue to emerge, it is becoming increasingly clear that this rat model of estrogen-induced mammary cancer and humans share multiple genetic determinants of breast cancer risk. The objectives of this study were to develop and characterize congenic rat strains to confirm the existence of *Emca3*, *Emca4*, *Emca5*, *Emca6*, *Emca7*, and *Emca9*, to quantify the effect of each of these QTL on mammary cancer development and to evaluate the relevance of these QTL to risk loci identified in GWAS. The animal models characterized in this study will serve as valuable resources for fine mapping and identifying the causal genetic variants that reside within each QTL as well as for defining the sites and mechanisms of action of these variants on mammary cancer development.

## Materials and Methods

### Care, treatment, and phenotypic characterization of animals

Studies presented herein were performed at two different institutions. All procedures involving live animals were approved by the respective Institutional Animal Care and Use Committees. ACI/SegHsd and BN/SsNHsd were obtained from Harlan Sprague Dawley, Inc. (Indianapolis, IN). The congenic rat strains used in this study were generated in our laboratory as described herein. All rats were housed under controlled temperature, humidity, and 12-hr light/12-hr dark conditions in facilities that were accredited by the American Association for Accreditation of Laboratory Animal Care and operated in accordance with *The Guide for the Care and Use of Laboratory Animals*. All procedures related to the care, propagation, genotyping, treatment with E2 and evaluation for presence of mammary cancer have been previously described (Shull *et al.* 1997; Spady *et al.* 1998; Harvell *et al.* 2000; Shull *et al.* 2001; Harvell *et al.* 2002; Gould *et al.* 2004; Gould *et al.* 2005; Schaffer *et al.* 2006; Kurz *et al.* 2008; Ding *et al.* 2013; Schaffer *et al.* 2013). The animals were generally killed after196 ± 5 days of treatment or earlier if necessitated because of tumor burden. However, 13 ACI rats were treated for up to 282 days. Raw latency and tumor number data for each rat strain are compiled in Supporting Information, Table S1.

### Generation and characterization of congenic rat strains

The congenic strains described herein were generated using a marker assisted selective breeding protocol as described previously (Schaffer *et al.* 2013). The markers used for positive and negative selection during backcrossing are listed in Table S2. Once a male rat was obtained that was heterozygous for BN alleles across a desired *Emca* locus and homozygous for ACI alleles at all background markers, that male was backcrossed to ACI females and sibling progeny carrying the same recombinant chromosome were intercrossed to produce rats that were homozygous for BN alleles across the specific *Emca* locus of interest (Table 1).

**Table 1:** Genetic characteristics of congenic strains

Strain Designation	Strain Abbreviation	Chr Length, Mb	Proximal ACI	Proximal BN	Distal BN	Distal ACI	RGD ID
ACI.BN-(*D2Rat251-D2Mgh3*)/Shul	ACI.BN-Emca3 or Emca3	258.2	*D2Mit29* 5.60 Mb	*D2Rat251* 6.33 Mb	*D2Mgh3* 63.00 Mb	*D2Rat22* 69.30 Mb	5687969
ACI.BN-(*D7Rat36-D7Rat11*)/Shul	ACI.BN-Emca4 or Emca4	143	Telomere	*D7Rat36* 1.53 Mb	*D7Rat11* 118.83 Mb	*D7Rat9* 125.40 Mb	6482654
ACI.BN-(*D3Rat80-D3Rat3*)/Shul	ACI.BN-Emca5 or Emca5	171	*D3Rat52* 14.05 Mb	*D3Rat80* 32.05 Mb	*D3Rat3* 162.58 Mb	*D3Rat59* 163.44 Mb	5688396
ACI.BN-(*D4Rat5-D4Got131*)/Shul	ACI.BN-Emca6 or Emca6	187.1	*D4Mgh22* 4.41 Mb	*D4Rat5* 9.63 Mb	*D4Got131* 162.28 Mb	*D4Mgh11* 171.20 Mb	5688400
ACI.BN-(*D6Rat148-D6Rat109*)/Shul	ACI.BN-Emca7 or Emca7	147.6	*D6Rat105* 18.62 Mb	*D6Rat148* 20.82 Mb	*D6Rat109* 146.16 Mb	Telomere	5688402
ACI.BN-(*D18Rat30-D18Rat89*)/Shul	ACI.BN-Emca9 or Emca9	87.3	Telomere	*D18Rat133* 3.55 Mb	*D18Rat89* 63.69 Mb	*D18Rat5* 77.45 Mb	5687973

BN, Brown Norway.

### Statistical analyses of data

MSTAT Version 5.4 was used to perform all statistical analyses (Drinkwater 2010). *P* values ≤ 0.05 were considered to be statistically significant. Latency was defined as the number of days separating initiation of E2 treatment and the first detection of a palpable mammary tumor. Median latency was derived from Kaplan-Meier analyses. The log rank test was used to compare latencies between strains and to calculate the relative risk of each set of congenic rats in comparison to ACI rats. The Wilcoxon rank sum test was used to compare the numbers of mammary tumors observed at necropsy. The susceptibilities of the different congenic rat strains to E2-induced mammary cancer were compared with ACI and BN rats evaluated contemporaneously at the same institution.

### Sources of genomic data

The locations of all genetic markers described herein were obtained from the Rat Genome Database and are based on Rat Genome Assembly, version 3.4 (Laulederkind *et al.* 2013; Nigam *et al.* 2013). Sequence variants between ACI and BN rats were obtained using the Variant Visualizer Tool available at Rat Genome Database. The lists of genes residing within each *Emca* locus were generated using the Biomart Tool at Ensembl and are based on Rat Genome Assembly version 3.4, Ensembl release 69, October 2012 (Kinsella *et al.*, 2011; Flicek *et al.* 2014). All references to genome coordinates in humans are based on genome assembly GRCh37.p10.

## Results and Discussion

### Validation and characterization of *Emca3*

**Figure 1:**
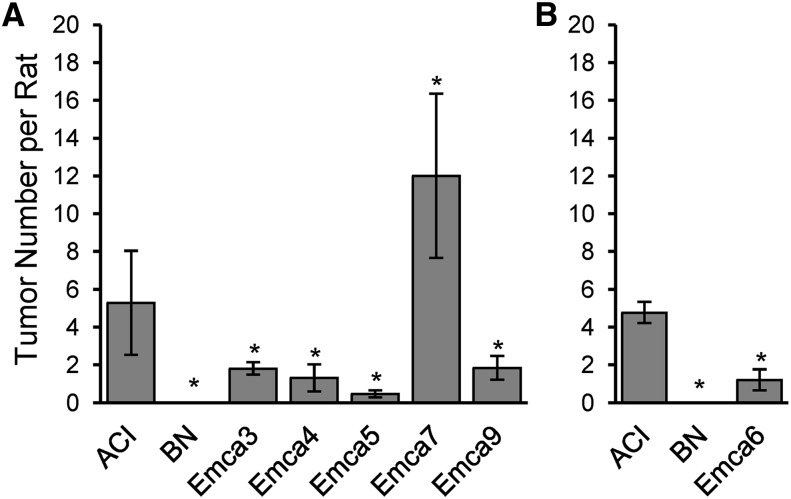
Effects of *Emca* loci on mammary cancer multiplicity. Beginning at 9 wk of age, female rats of each of the indicated rat strains were treated with E2 released continuously from subcutaneous Silastic implants. Female rats from each congenic strain were evaluated contemporaneously with batches of ACI and BN rats. Because the mammary cancer phenotypes exhibited by susceptible ACI and resistant BN rats at each of the two institutions were highly reproducible, the individual batches of ACI and BN rats evaluated at each institution were pooled for comparison with congenic rats evaluated at the same institution. (A) Animals evaluated at the University of Nebraska Medical Center. (B) Animals evaluated at the University of Wisconsin-Madison. Each data bar indicates the mean tumor number (± SEM) observed at necropsy for rats treated for at least 160 days and no more than 201 days; n = 10−83 animals per group. Asterisks indicate statistical significance (*P* < 0.05) compared with ACI females evaluated at the same institution.

**Figure 2:**
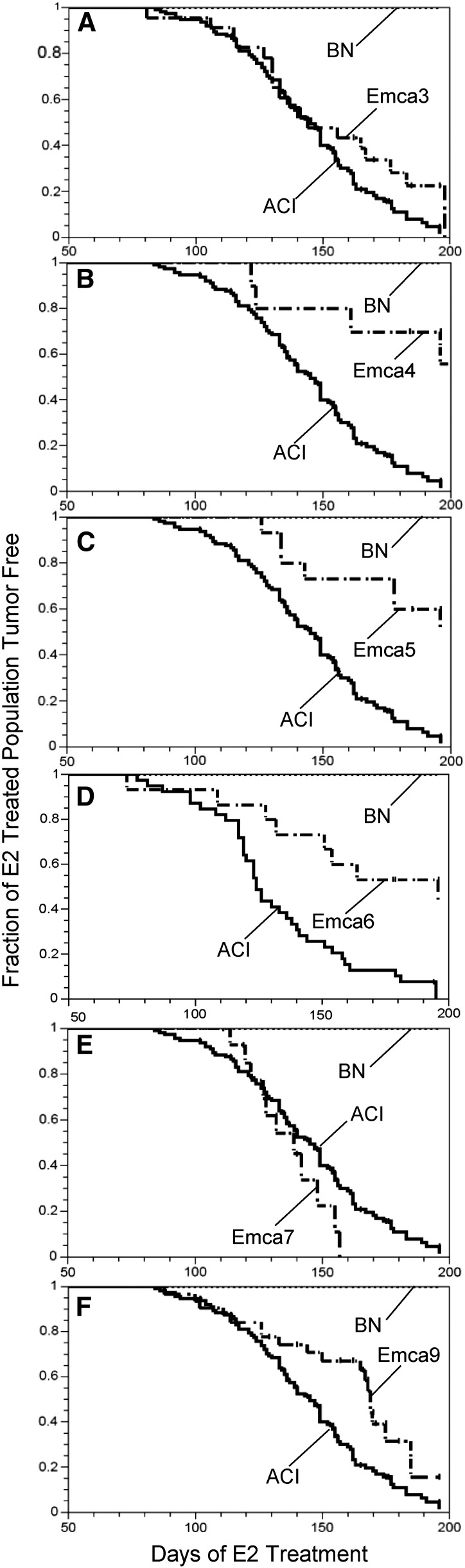
Effects of *Emca* loci on mammary cancer latency and incidence. Female rats of each of the indicated rat strains were treated with E2 as described in the section *Materials and Methods* and Figure 1 and were evaluated by palpation once or twice weekly to detect the presence of mammary cancer. Each data panel illustrates the number of days separating initiation of E2 treatment and detection of palpable mammary cancer or grossly apparent mammary cancer at necropsy. (A) ACI.BN-Emca3, (B) ACI.BN-Emca4, (C) ACI.BN-Emca5, (D) ACI.BN-Emca6, (E) ACI.BN-Emca7, and (F) ACI.BN-Emca9. Congenic rat strains were compared with populations of E2-treated ACI and BN rats evaluated at the same institution. Sample sizes ranged from 11 to 126 rats per strain.

*Emca3* was localized to RNO2 in an interval mapping study of F2 progeny generated in an intercross between ACI females and BN males (B. Schaffer and J. Shull, unpublished data). The peak LOD (logarithm of odds ratio) region for *Emca3* was nearest *D2Rat16* (43.4 Mb), although the LOD peak for this QTL did not achieve genome-wide significance (Table 3). To evaluate this putative QTL further, the ACI.BN-Emca3 congenic rat strain was generated by introgressing BN alleles from *D2Rat251* (6.3Mb) to *D2Mgh3* (63.0Mb) onto the ACI genetic background (Table 1). Based on the interval mapping data, it was hypothesized that ACI.BN-Emca3 congenic rats would exhibit reduced susceptibility to E2-induced mammary cancer when compared with ACI rats. ACI rats developed an average of 5.3 ± 4.5 (mean ± SD) mammary cancers when treated with E2 (Figure 1A and Table 2 illustrate data on group sizes). By contrast, treated ACI.BN-Emca3 rats developed only 1.8 ± 1.5 cancers per rat (*P* < 0.0001 compared with E2-treated ACI rats). Latency to appearance of palpable mammary cancer did not differ between E2-treated ACI and ACI.BN-Emca3 rats (Figure 2A and Table 2). Data from E2-treated BN rats, which are highly resistant to E2-induced mammary cancer and did not develop mammary cancers within the 196-day course of treatment, are illustrated for comparison. These data confirm the existence of *Emca3* on RNO2.

**Table 2:** Mammary cancer phenotypes

Strain	Median Latency, d*^a^*	Latency P* *vs.* ACI*^b^*	Hazard Ratio*^b^*	Mean Latency, d*^c^*	Incidence, %*^d^*	Mean Tumor Number per Rat*^e^*	Tumor Number P* *vs.* ACI*^f^*	N*^g^*
ACI (UNMC)	147	−	1.000	140 ± 27	94	5.3 ± 4.5	−	126 (83)
BN (UNMC)	−	2.66e-6	0.000	−	0	0	1.08e-6	10 (10)
ACI.BN-Emca3	144	0.2280	0.7358	141 ± 29	100	1.8 ± 1.5	0.0001	23 (20)
ACI.BN-Emca4	NA*^h^*	0.0017	0.2322	151 ± 35	43	1.3 ± 2.3	0.0004	11 (10)
ACI.BN-Emca5	NA*^h^*	0.0003	0.2704	156 ± 28	48	0.5 ± 0.7	6.17e-7	15 (13)
ACI.BN-Emca7	139	0.1153	1.640	135 ± 14	100	12 ± 5.9	0.0002	15 (11)
ACI.BN-Emca9	169	0.2251	0.7327	142 ± 30	84	1.8 ± 3.0	5.13e-6	32 (23)
ACI (UW)	126	−	1.000	128 ± 25	100	4.8 ± 3.6	−	45 (42)
BN (UW)	−	8.39e-6	0.000	−	0	0	1.19e-5	10 (9)
ACI.BN-Emca6	196	0.0010	0.3027	138 ± 37	56	1.2 ± 2.1	8.82e-5	15 (15)

UNMC, University of Nebraska Medical Center; BN, Brown Norway; NA, not applicable; UW, University of Wisconsin.

a Calculated from Kaplan-Meier analysis.

b Calculated using log rank test.

c Calculated for tumor positive rats (mean ± SD).

d Calculated for population at risk.

e Calculated for all rats that were treated with E2 ≥160 days but ≤200 days (mean ± SD).

f Calculated using Wilcoxon rank sum test.

g Total number of rats treated with E2 (total number of rats treated ≥160 days but ≤200 days.)

h Not applicable. Median latency exceeds 196 days.

* *P* ≤ 0.05 indicates statistical significance.

The *Emca3* locus is orthologous to two distinct regions of human chromosome (Chr) 5, 5p12 and 5q11.2, and multiple single-nucleotide polymorphisms (SNPs) residing within these regions of Chr5 have been associated with breast cancer (Table 3). Positional candidate genes residing near the Chr5 breast cancer associated SNPs and/or suggested to mediate the actions of the causal genetic variants include *MRPS30*, *HCN1*, *MAP3K1*, *MIER3*, *RAB3C*, and *PDE4D* (Easton *et al.* 2007; Stacey *et al.* 2008; Thomas *et al.* 2009; Turnbull *et al.* 2010; Michailidou *et al.* 2013). Moreover, three studies suggest that mammographic breast density, a well-established biomarker of breast cancer risk, is influenced by genetic variants that reside within this same region of the human genome, near *MRPS30*, *HCN1*, and *MAP3K1* (Table 4) (Woolcott *et al.* 2009; Odefrey *et al.* 2010; Fernandez-Navarro *et al.* 2013). Each of these genes that have been associated with breast cancer risk and/or mammographic density resides within the *Emca3* peak LOD region on RNO2. Taken together, the data from the rat models and humans suggest that the causal variants responsible for the actions of *Emca3* on mammary cancer susceptibility may reside on RNO2 within the maximal interval defined by the individual GWAS; *i.e.*, *Pde4d* (40.1 Mb) to *Mrps30* (50.6 Mb). This region contains 55 known protein coding genes, including the six noted above (Table S3). Evaluation of whole-genome sequences for the ACI and BN rat strains revealed nonsynonymous coding region variants within several genes residing within *Emca3*, including *Map3k1* (2 nonsynonymous variants), *Ndufs4*, *Mocs2*, *Hcn1*, and *Mrps30* (Table S4). The impact of these variants on the functions of their respective proteins and mammary cancer susceptibility is not known. *Emca3* also harbors several genes that encode small RNAs, including *miR449a*, *miR449c*, and *miR582* (Table S3). *Mcs1*, a complex QTL that determines susceptibility to mammary cancers induced by the polycyclic aromatic hydrocarbon dimethylbenz[*a*]anthracene in backcross progeny generated in crosses between susceptible Wistar-Furth rats and resistant COP rats, has been mapped to the same region of RNO2 as *Emca3* (Hsu *et al.* 1994; Shepel *et al.* 1998; Haag *et al.* 2003; DenDekker *et al.* 2012). Together, these data suggest that genetic variants residing within the *Emca3* and *Mcs1* loci on RNO2 may influence development of mammary cancer in response to both estrogens and the genotoxic carcinogen dimethylbenz[*a*]anthracene.

**Table 3:** Orthologous relationships between *Emca* susceptibility loci in rat and breast cancer risk loci in humans

Locus	Peak LOD Marker	Proximal 95% Confidence Interval Marker	Distal 95% Confidence Interval Marker	Human Locus	SNP	Chr. Position of Human SNP	Gene	Orthologous Rat Position, Mb	Reference
*Emca3*	*D2Rat16*	−	−	5q11	rs1353747	58337481	PDE4D	41.3	Michailidou *et al.* 2013
	43.4 Mb			5q11	rs10472076	58184061	RAB3C	41.4	Michailidou *et al.* 2013
				5q11	rs889312	56031884	MAP3K1, MIER3	43.2	Easton *et al.* 2007; Turnbull *et al.* 2010
				5q11	rs16886165	56023083	MAP3K1	43.2	Thomas *et al.* 2009
				5q11	rs30099	52418582	−	46.9	Easton *et al.* 2007
				5p12	rs981782	45285718	−	49.9	Easton *et al.* 2007
				5p12	rs2067980	44982317	MRPS30	50.3	Thomas *et al.* 2009
				5p12	rs7716600	44875005	MRPS30	50.5	Thomas *et al.* 2009
				5p12	rs10941679	44706498	MRPS30, HCN1	50.6	Stacey *et al.* 2008; Thomas *et al.* 2009
*Emca4*	*D7Rat19*	*D7Rat44*	*D7Rat15*	8q24	rs13281615	128355618	MYC	98.4	Easton *et al.* 2007
	98.6 Mb	66.2 Mb	107.4 Mb	8q24	rs1562430	128387852	MYC	98.5	Thomas *et al.* 2009; Turnbull *et al.* 2010
				8q24	rs11780156	129194641	MIR1208	99.3	Michailidou *et al.* 2013
*Emca5*	*D3Rat114*	*D3Rat227*	*D3Rat210*	2q31	rs2016394	172972971	METAP1D	53.9	Michailidou *et al.* 2013
	149.5 Mb	41.1 Mb	156.6 Mb	2q31	rs1550623	174212894	CDCA7	54.9	Michailidou *et al.* 2013
				8p12	rs9693444	29509616	−	63.2	Michailidou *et al.* 2013
				20q11	rs2284378	32588095	RALY	145.3	Siddiq *et al.* 2012
*Emca6*	*D4Rat103*	*D4Rat14*	*D4Rat202*	7q35	rs720475	144074929	ARHGEF5, NOBOX	71.1	Michailidou *et al.* 2013
	82.7Mb	41.7Mb	159.1 Mb	3p26	rs6762644	4742276	ITPR1, EGOT	143.9	Michailidou *et al.* 2013
*Emca7*	*D6Rat22*	*D6Rat68*	*D6Rat81*	2p24	rs4666451	19286943	−	33.3	Easton *et al.* 2007
	75.5 Mb	2.8 Mb	112.0 Mb	2p24	rs12710696	19320803	−	33.3	Garcia-Closas *et al.* 2013
				14q13	rs2236007	37132769	PAX9, SLC25A21	77.1	Michailidou *et al.* 2013
				14q24	rs2588809	68660428	RAD51L1	102.5	Michailidou *et al.* 2013
				14q24	rs999737	69034382	RAD51B	103.9	Thomas *et al.* 2009
				14q32	rs941764	91841069	CCDC88C	125.3	Michailidou *et al.* 2013
*Emca8*	*D5Rat95*	*D5Rat134*	*D5Rat37*	9q31	rs10759243	110306115	−	73.5	Michailidou *et al.* 2013
	130.6 Mb	52.43 Mb	148.5 Mb	9p21	rs1011970	22062134	CDKN2A, CDKN2B	109.0	Turnbull *et al.* 2010
				1p36	rs616488	10566215	PEX14	166.1	Michailidou *et al.* 2013
*Emca9*	*D18Rat30*	−	−	18q11	rs527616	24337424	−	6.5	Michailidou *et al.* 2013
	5.7 Mb			18q11	rs1436904	24570667	CHST9	6.8	Michailidou *et al.* 2013

LOD, logarithm of odds ratio; SNP, single-nucleotide polymorphism.

**Table 4:** Orthologous relationship between *Emca* susceptibility loci in rat and breast density loci in humans

Locus	Peak Marker	Proximal 95% Confidence Interval Marker	Distal 95% Confidence Interval Marker	Human Locus	SNP	Chr. Position of Human SNP	Gene	Orthologous Rat Position, Mb	Reference
*Emca3*	*D2Rat16*	−	−	5q11	rs889312	56031884	MAP3K1, MIER3	43.2	Odefrey *et al.* 2010
	43.4 Mb			5p12	rs10941679	44706498	HCN1, MRPS30	50.6	Woolcott *et al.* 2009
				5p12	rs4415084	44662515	MRPS30	69.5	Fernandez-Navarro *et al.* 2013
*Emca4*	*D7Rat19* 98.6 Mb	*D7Rat44* 66.2 Mb	*D7Rat15* 107.4 Mb	8q24	rs13281615	128355618	MYC	98.4	Odefrey *et al.* 2010
*Emca6*	*D4Rat103* 82.7 Mb	*D4Rat14* 41.7Mb	*D4Rat202* 159.1 Mb	7q34	rs4728251	131858336	RAB19	59.3	Greenwood *et al.* 2011
*Emca7*	*D6Rat22* 75.5 Mb	*D6Rat68* 2.8 Mb	*D6Rat81* 112.0 Mb	14q24.1	rs10483813	69031284	RAD51L1	102.9	Vachon *et al.* 2012

SNP, single-nucleotide polymorphism.

### Validation and characterization of *Emca4*

*Emca4* was mapped to RNO7 in an interval mapping study of F2 progeny generated in a BN × ACI intercross (Schaffer *et al.* 2006). The LOD peak for this QTL was near *D7Rat19* (98.6 Mb) and the 95% confidence interval extended from *D7Rat44* (66.2Mb) to *D7Rat15* (107.4Mb) (Table 3). The ACI.BN-Emca4 congenic strain was generated by introgressing BN alleles from *D7Rat36* (1.53Mb) to *D7Rat11* (118.83Mb) onto the ACI genetic background (Table 1). ACI.BN-Emca4 rats exhibited significantly reduced susceptibility to E2-induced mammary cancer relative to ACI rats, thereby confirming the published mapping data (Table 2). Tumor number at termination of the study was lower in ACI.BN-Emca4 rats than in ACI rats; 1.3 ± 2.3 tumors per rat *vs.* 5.3 ± 4.5, *P* = 0.0004 (Figure 1A and Table 2). Moreover, latency to appearance of palpable mammary cancer was prolonged in ACI.BN-Emca4 rats, relative to ACI rats, and only 43% of ACI.BN-Emca4 rats at risk developed mammary cancer within the 196 day course of E2 treatment, compared to 94% for E2 treated ACI rats (Figure 2B and Table 2).

The peak LOD region of *Emca4* is orthologous to the Chr8q24 breast cancer risk locus in humans identified in multiple GWAS (Easton *et al.* 2007; Thomas *et al.* 2009; Turnbull *et al.* 2010; Michailidou *et al.* 2013). A genetic determinant of mammographic breast density has also been mapped to the 8q24 locus (Odefrey *et al.* 2010). The 8q24 breast cancer risk locus is defined by a gene desert that surrounds the *MYC* proto-oncogene. *Myc* similarly resides at the *Emca*4 LOD peak (Schaffer *et al.* 2006). It has been hypothesized that genetic variants in the 8q24 risk locus reside within *cis*-acting regulators of transcription, impact *MYC* expression in a cell type specific or temporal manner, and thereby modify breast cancer risk (Ahmadiyeh *et al.* 2010). Together, the data from the rat models and humans suggest an evolutionarily conserved determinant of breast cancer risk resides within *Emca4* near *Myc*. Table S5 and Table S6 list the genes and variants, respectively, residing within the 10-Mb region of RNO7 centered on *Myc*. The 8q24 locus is pleiotropic with respect to its influence on disease susceptibility and has been associated with multiple cancer types in addition to breast cancer, including ovarian, prostate, colorectal, and bladder cancers (Braem *et al.* 2011; Ishak and Giri 2011; Theodoratou *et al.* 2012; Ma *et al.* 2013; Sur *et al.* 2013). *Ept7*, a QTL that impacts development of E2-induced pituitary tumors in (BNxACI)F2 rats, has been mapped to the same region of RNO7 as *Emca4* (Shull *et al.* 2007; Kurz *et al.* 2014). Together, these data suggest the possibility that the causal variant(s) residing within the overlapping *Emca4* and *Ept7* loci may exert pleiotropic effects on tumor development in multiple estrogen responsive tissues. We are fine mapping *Emca4* and *Ept7* to identify the causal genetic variants that determine susceptibility to mammary cancers and pituitary tumors and to define the sites and mechanisms of action of these causal variants.

### Validation and characterization of *Emca5*

Interval mapping analyses of (BNxACI)F2 progeny localized *Emca5* to RNO3 (Schaffer *et al.* 2006). The LOD peak for this QTL was near *D3Rat114* (149.5 Mb) and the 95% confidence interval extended from D3Rat227 (41.1 Mb) to near D3Rat210 (156.6 Mb) (Table 3). The ACI.BN-Emca5 congenic strain was generated by introgressing BN alleles from *D3Rat80* (32.1 Mb) to *D3Rat3* (162.6 Mb) onto the ACI genetic background (Table 1). ACI.BN-Emca5 rats exhibited dramatically reduced susceptibility to E2-induced mammary cancer, relative to ACI rats (Table 2). Tumor number at the termination of the experiment was significantly reduced (Figure 1A, Table 2), latency to appearance of palpable mammary cancer was significantly prolonged, and mammary cancer incidence was reduced in E2-treated ACI.BN-Emca5 rats compared with treated ACI rats (Figure 2C, Table 2).

The peak LOD region of *Emca5* is orthologous to a segment of the long arm of Chr20q11 that has been associated with breast cancer risk (Siddiq *et al.* 2012). RALY has been identified as a candidate gene for this risk locus, and the rat ortholog of *Raly* resides at 145.3 Mb on RNO3, the peak LOD region for *Emca5*. The 20-Mb interval of RNO3 that extends from 135 to 155 Mb and flanks the peak LOD region of *Emca5* harbors more than 250 annotated genes (Table S7). No functionally significant variants were identified in *Raly* upon comparison of available ACI and BN genome sequences, although nonsynonymous variants were identified in other genes residing within the *Emca5* region (Table S8). It is also noteworthy that the proximal shoulder of *Emca5* is orthologous to two additional regions of the human genome that have been linked to breast cancer risk in humans, Chr2q31 and Chr8p12 (Michailidou *et al.* 2013). Together, these data confirm the existence of *Emca5* as a genetic determinant of susceptibility to E2-induced mammary cancer in the rat and suggest that *Emca5* may harbor one or more genetic determinants of mammary cancer susceptibility that are shared with humans. Identification of *Emca5* candidates for further evaluation will require this locus to be mapped more precisely using additional congenic rat strains.

### Validation and characterization of *Emca6*

*Emca6* was defined in (BNxACI)F2 rats as a QTL with a LOD peak located proximal to *D4Rat103* (82.7 Mb) and a 95% confidence interval spanning from *D4Rat14* (41.7 Mb) to *D4Rat202* (159.1 Mb) (Table 3) (Schaffer *et al.* 2006). The ACI.BN-Emca6 congenic rat strain harbors BN alleles spanning from *D4Rat5* (9.6 Mb) to *D4Got131* (162.3 Mb) introgressed onto the ACI genetic background (Table 1). As predicted from the published interval mapping data, ACI.BN-Emca6 rats were less susceptible than ACI rats to E2-induced mammary cancer. Mammary tumor number was lower (Figure 1B and Table 2), latency to appearance of mammary cancer was prolonged and incidence of mammary cancer was reduced (Figure 2D and Table 2) in E2 treated ACI.BN-Emca6 rats when compared to treated ACI rats. These data confirm the existence of the *Emca6* mammary cancer QTL.

The peak LOD region of *Emca6* is orthologous to a human breast cancer risk locus at Chr7q35, tagged by SNP rs720475 and harboring *ARHGEF5* and *NOBOX* (Michailidou *et al.* 2013). In the rat, these genes reside at 71.1 Mb on RNO4 (Table 3 and Table S9). Comparison of genome sequences for ACI and BN rats revealed multiple variants within *Arhgef5* and *Nobox*; however, none of these occur in conserved positions suggesting they may not be of functional significance (Table S10). The peak LOD region of *Emca6* is also orthologous to an interval on Chr7q34 containing RAB19 that has been associated with mammographic density in premenopausal women (Greenwood *et al.* 2011) (Table 4). *Rab19* in rat resides at 66.9 Mb on RNO4. The 20 Mb interval centered on the *Emca6* LOD peak contains 221 annotated protein coding genes and 21 genes that encode various classes of RNA (Table S9). Genes within this interval that harbor nonsynonymous variants include *Trim24*, which encodes an estrogen receptor-alpha interacting protein that has been suggested to contribute to breast cancer development (Tsai *et al.* 2010) and *Hoxa7*, which encodes a transcription factor that regulates expression of estrogen receptor-alpha (Zhang *et al.* 2013). In addition, a more distal segment of *Emca6* is orthologous to a second human breast cancer risk locus at Chr3p26 that harbors *ITPR1* and *EGOT* (Michailidou *et al.* 2013). *Itpr1* resides at position 143.7 Mb on RNO4. *Egot*, a non-protein encoding gene, is not annotated in rat.

### Validation and characterization of *Emca7*

*Emca7* was mapped to RNO6 in a BN × ACI intercross (Schaffer *et al.* 2006). The LOD peak for this QTL resided proximal to *D6Rat22* (75.5 Mb) and the 95% confidence interval extended from *D6Rat68* (2.8 Mb) to *D6Rat81* (112.0 Mb) (Table 3). *Emca7* was unique among the *Emca* loci mapped in reciprocal intercrosses between susceptible ACI rats and resistant BN rats in that BN alleles at *Emca7* were associated with increased susceptibility to E2-induced mammary cancer. ACI.BN-Emca7 congenic rats harbor BN alleles from *D6Rat148* (20.8 Mb) to *D6Rat109* (146.2 Mb) introgressed on the ACI genetic background (Table 1). When treated with E2, ACI.BN-Emca7 rats exhibited increased susceptibility to mammary cancer, relative to ACI rats. The number of mammary tumors observed at termination of the experiment was significantly higher in E2-treated ACI.BN-Emca7 rats compared with that observed in treated ACI rats (Figure 1A and Table 2). However, latency to appearance of palpable mammary cancer did not differ significantly upon comparison of ACI.BN-Emca7 and ACI rats (Figure 2E and Table 2).

Three distinct regions within *Emca7* are orthologous to breast cancer risk loci identified in GWAS (Table 3). The region of RNO6 at 33.3 Mb is orthologous to a risk locus at Chr2p24 tagged by SNPs rs4666451 and rs12710696 (Easton *et al.* 2007; Garcia-Closas *et al.* 2013). The region of RNO6 near *D6Rat22* (75.5 Mb), the marker nearest the *Emca7* LOD peak, is orthologous to a breast cancer risk locus at Chr14q13, which harbors two suggested candidate genes *PAX9* and *SLC25A21* (77.1 Mb on RNO6) (Michailidou *et al.* 2013). Finally, the distal segment of the *Emca7* confidence interval is orthologous to a risk locus at Chr14q24, which harbors two candidate genes *RAD51B* and *RAD51L1* (102.1 Mb on RNO6) (Thomas *et al.* 2009; Michailidou *et al.* 2013). This region of Chr14q24 has also been associated with mammographic breast density (Vachon *et al.* 2012) (Table 4). The genes and variants that reside within 20 Mb of the LOD peak for *Emca7* are listed in Table S11 and Table S12, respectively.

### Validation and characterization of *Emca9*

*Emca9* was identified in an intercross between ACI and BN rats as a suggestive QTL on RNO18 with a LOD peak at *D18Rat30* (5.7 Mb) (Table 3) (B. Schaffer and J. Shull, unpublished data). To confirm the existence of this QTL, the ACI.BN-Emca9 congenic strain was generated by introgressing onto the ACI genetic background BN alleles from *D18Rat133* (3.6 Mb) to *D18Rat89* (63.7 Mb) (Table 1). Compared with ACI rats, ACI.BN-Emca9 congenic rats exhibited reduced susceptibility to E2-induced mammary cancer as evidenced by fewer mammary tumors and a lower mammary cancer incidence upon termination of the experiment (Figure 1A and Table 2). Latency to appearance of palpable mammary cancer did not differ significantly between E2-treated ACI and ACI.BN-Emca9 rats (Figure 2F and Table 2).

The peak LOD region of *Emca9* is orthologous to Chr18q11, which harbors two distinct breast cancer risk loci, the latter of which contains the candidate gene *CHST9* (Table 3) (Michailidou *et al.* 2013). The proximal 15 Mb of RNO18, which constitutes the peak LOD region for *Emca9*, harbors 71 annotated protein coding genes (Table S13). Comparison of whole-genome sequences for ACI and BN rats revealed three nonsynonymous variants in *Chst9* (Table S14). These data confirm the existence of *Emca9* as a genetic determinant of susceptibility to E2-induced mammary cancer and suggest that this QTL may be orthologous to a breast cancer risk locus in humans. Finally, it is noted that *Emca9* overlaps with *Emca2*, a genetic determinant of susceptibility to E2-induced mammary cancer that was mapped to RNO18 in an intercross between susceptible ACI rats and resistant COP rats (Gould *et al.* 2004).

### Sham-treated control rats do not develop mammary cancer

Small groups of ACI, BN, and ACI.BN-Emca congenic rats (n = 4−7 rats per strain) received empty Silastic implants and were evaluated as sham-treated contemporaneous controls. No mammary cancers were observed in sham-treated control ACI or BN rats or in control female rats from any of the ACI.BN-Emca congenic strains described herein. These data indicate that development of mammary cancers in the ACI and the different strains of ACI.BN-Emca congenic rats requires treatment with E2, and are consistent with previous studies that indicate that ACI and congenic rat strains established on the ACI genetic background are resistant to spontaneously arising mammary cancer (Maekawa and Odashima 1975; Shull *et al.* 1997; Shull *et al.* 2001; Gould *et al.* 2004; Schaffer *et al.* 2006; Kurz *et al.* 2008; Schaffer *et al.* 2013).

Six unique congenic rat strains were generated and evaluated in this study to define further the genetic bases of susceptibility to mammary cancer in a rat model in which tumor development is induced by treatment with E2, a naturally occurring steroid hormone that has been inextricably implicated in breast cancer development. The data presented herein provide the first published evidence of the existence of *Emca3* on RNO2 and *Emca9* on RNO18 and confirm the existence of *Emca4* on RNO7, *Emca5* on RNO3, *Emca6* on RNO4, and *Emca7* on RNO6. A similar congenic rat based approach was used recently to confirm the existence of *Emca8* on RNO5 (Schaffer *et al.* 2013). Therefore, the existence of all seven of the *Emca* QTL mapped previously in intercrosses between susceptible ACI rats and resistant BN rats has now been independently verified.

Data presented herein also indicate that the peak LOD region for each of the 6 *Emca* loci characterized in this study is genetically orthologous to one or more genetic determinants of breast cancer risk mapped in GWAS. The orthologous relationship between the peak LOD region of *Emca8* and the Chr9p21 breast cancer risk locus in humans was noted previously (Schaffer *et al.* 2013). Moreover, a recently published GWAS identified a breast cancer risk locus at Chr9q31 that is orthologous to a second local LOD peak within the complex *Emca8* locus (Michailidou *et al.* 2013; Schaffer *et al.* 2013). Together, these data strongly suggest that the rat and human share multiple genetic determinants of mammary cancer susceptibility. By confirming the existence of seven distinct *Emca* loci and illustrating their genetic relatedness to breast cancer risk loci in humans, the present and recently published studies further establish the relevance of the ACI and ACI.BN-Emca congenic rat models of E2-induced mammary cancer to breast cancer in humans.

Interestingly, the peak LOD regions of *Emca3*, *Emca4*, *Emca6* and the distal segment of *Emca7* are orthologous to regions of the human genome that have been associated with mammographic breast density, a biomarker of breast cancer risk (Woolcott *et al.* 2009; Odefrey *et al.* 2010; Greenwood *et al.* 2011; Vachon *et al.* 2012; Fernandez-Navarro *et al.* 2013). Mammographic density is determined by the relative amounts of radiodense epithelium and stroma relative to the amount of radiolucent adipose tissue. Multiple cellular and molecular phenotypes have been identified that differ quantitatively and/or qualitatively between mammary glands of E2-treated ACI and BN rats, including epithelial density, which is greater in susceptible ACI rats, and luminal ectasia and associated collagenous stroma, which are more prominent in mammary glands of resistant BN rats (Ding *et al.* 2013). These data suggest that some radiodense features, *e.g.*, epithelial density, may segregate with susceptibility conferring alleles, whereas others, *e.g.*, luminal ectasia and associated collagenous stroma, may segregate with resistance conferring alleles. If true, then development of imaging technologies that can discriminate these specific phenotypes may enhance the value of breast imaging for breast cancer risk prediction.

The data emerging from studies focused on identification of low penetrance determinants of breast cancer risk strongly suggest that the causal genetic variants, once identified, will likely prove to be noncoding nucleotide or structural variants that influence expression of specific genes in a cell type specific and/or temporal manner. Moreover, the actions of the causal genetic variants on breast cancer risk may be extrinsic to the mammary epithelial cell population in which breast cancers arise. These considerations impose limits upon the use of breast cancer cell lines and human subjects for identifying the causal variants and establishing their functional roles in breast cancer etiology. The ACI.BN-Emca congenic rat strains described herein will serve as valuable models for identifying the causal genetic variants that influence susceptibility to E2-induced mammary cancer as well as for identifying the cell type(s) in which these variants exert their effects and defining the molecular mechanisms through which these actions are conferred. The knowledge to be generated by further characterization of these rat models should significantly enhance our understanding of the genetic bases underlying breast cancer risk as well as the mechanisms through which estrogens contribute to breast cancer development.

## Supplementary Material

Supporting Information
