# Supplementary material for: Validation of Six Genetic Determinants of Susceptibility to Estrogen-Induced Mammary Cancer in the Rat and Assessment of Their Relevance to Breast Cancer Risk in Humans
Source: G3 (Bethesda). 2014 May 28;4(8):1385–94. doi: 10.1534/g3.114.011163 (PMC4132170; doi:10.1534/g3.114.011163)
Supplement: Supporting Information [file supp_4_8_1385__index.html]

Validation of Six Genetic Determinants of Susceptibility to Estrogen-Induced Mammary Cancer in the Rat and Assessment of Their Relevance to Breast Cancer Risk in Humans — Supporting Information 

# Validation of Six Genetic Determinants of Susceptibility to Estrogen-Induced Mammary Cancer in the Rat and Assessment of Their Relevance to Breast Cancer Risk in Humans

## Supporting Information for Colletti *et al.*, 2014

**Files in this Data Supplement:**

- Table S1 - Raw latency and tumor number data collected for rat strains ACI/SegHsd, BN/SsNHsd, ACI.BN-Emca3, ACI.BN-Emca4, ACI.BN-Emca5, ACI.BN-Emca6, ACI.BN-Emca7 and ACI.BN-Emca9. (.xlsx, 27 KB)
- Table S2 - Markers defining quantitative trait loci *Emca3, Emca4, Emca5, Emca6, Emca7* and *Emca9*. (.xlsx, 16 KB)
- Table S3 - Genes associated with quantitative trait locus *Emca3*. (.xlsx, 21 KB)
- Table S4 - Variants associated with quantitative trait locus *Emca3*. (.xlsx, 1 MB)
- Table S5 - Genes associated with quantitative trait locus *Emca4*. (.xlsx, 22 KB)
- Table S6 - Variants associated with quantitative trait locus *Emca4*. (.xlsx, 581 KB)
- Table S7 - Genes associated with quantitative trait locus *Emca5*. (.xlsx, 76 KB)
- Table S8 - Variants associated with quantitative trait locus *Emca5*. (.xlsx, 899 KB)
- Table S9 - Genes associated with quantitative trait locus *Emca6*. (.xlsx, 57 KB)
- Table S10 - Variants associated with quantitative trait locus *Emca6*. (.xlsx, 1 MB)
- Table S11 - Genes associated with quantitative trait locus *Emca7*. (.xlsx, 64 KB)
- Table S12 - Variants associated with quantitative trait locus *Emca7*. (.xlsx, 3 MB)
- Table S13 - Genes associated with quantitative trait locus *Emca9*. (.xlsx, 28 KB)
- Table S14 - Variants associated with quantitative trait locus *Emca9*. (.xlsx, 1 MB)
